# Severe Relapsing Goodpasture's Disease Successfully Treated with Mycophenolate Mofetil

**DOI:** 10.4061/2010/383548

**Published:** 2010-08-16

**Authors:** Anabela Malho, Viriato Santos, Ana Cabrita, Ana Paula Silva, Isabel Pinto, Idalécio Bernardo, Pedro Leão Neves

**Affiliations:** Serviço de Nefrologia, Hospital de Faro, Rua Leão Penedo, 8000 Faro, Portugal

## Abstract

Goodpasture's disease is a rare autoimmune disorder characterised by the development of antiglomerular basement membrane autoantibodies, which typically presents with rapidly progressive crescentic glomerulonephritis and pulmonary haemorrhage. Even with aggressive nonspecific immunosuppression and plasma exchange, mortality remains high. We report a case of life-threatening Goodpasture's disease with relapsing pulmonary haemorrhage refractory to conventional therapy. Second line treatment was based on mycophenolate mofetil 1 g every 12 hours and prednisolone 60 mg/day. In this case, the use of a low-dose mycophenolate mofetil regimen turned out to be insufficient. However, in our opinion higher mycophenolate mofetil doses should be considered an alternative treatment, mainly in relapsing disease, due to its mechanism of action and current insufficient therapeutic weapons.

## 1. Introduction

Goodpasture's disease is an autoimmune disorder characterised by the development of autoantibodies to the NC1 domain of the *α*3 chain of type IV collagen, found in glomerular, pulmonary, and other basement membranes. Clinical presentation comprises rapidly progressive crescentic glomerulonephritis and pulmonary haemorrhage [1, 2]. It is an uncommon disease estimated to occur in less than one case per million population [3]. Without treatment it is a frequently fatal disease. Conventional therapy is based on a combination of plasma exchange with aggressive nonspecific immunosuppression and relapses are uncommon [4]. Despite this, mortality remains high, with median time to death two months in patients with pulmonary haemorrhage [5, 6]. We report a case of life-threatening relapsing Goodpasture's syndrome refractory to conventional therapy which responded to treatment with mycophenolate mofetil.

## 2. Case Report

**Figure 1:**
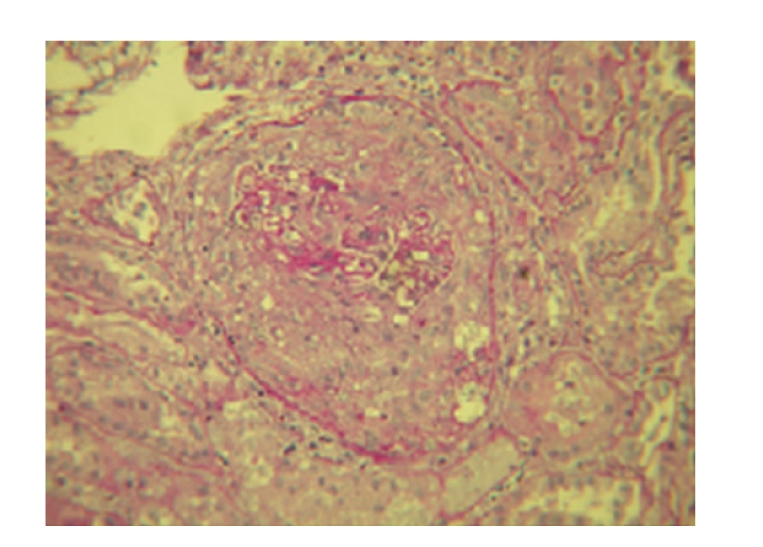
Light microscopy photograph (PAS: 250×): Circumferential cellular crescent with compressed capillary tuft.

**Figure 2:**
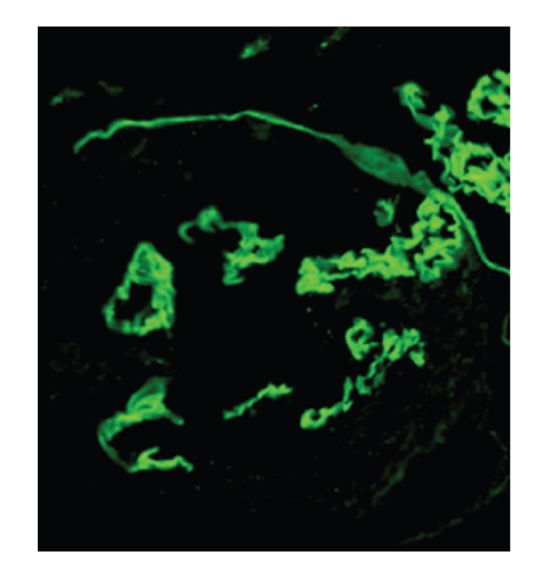
Immunofluorescence microscopy photograph (IgG: 250×): Linear IgG deposits in glomerular basement membrane with Bowman capsule rupture.

A 19-year-old man presented with cough and haemoptysis, evolving with severe anaemia, dyspnoea, haematuria, and oliguric rapidly progressive renal failure three weeks later. The patient had a history of smoking and had inhaled cocaine weeks before the complaints onset. On admission he was pale, with tachypnea, tachycardia, and elevated blood pressure. Laboratory results revealed haematocrit 20%; haemoglobin 7.0 g/dL; blood urea nitrogen 81 mg/dL; serum creatinine 7.4 mg/dL; albumin 3.3 g/dL and potassium 4.6 mmol/L. Complement components and serum immunoglobulins were normal, antinuclear antibodies, antiDNA antibodies, immune complexes, cryoglobulins, and antineutrophil cytoplasmatic autoantibodies were negative while anti-GBM antibodies were positive. Chest X-ray bilateral alveolar shadowing was indicative of pulmonary haemorrhage. A renal biopsy specimen (Figures 1 and 2) showed an extracapillary glomerulonephritis with cellular crescents in five out of six glomeruli, mild diffuse interstitial oedema, mild tubular atrophy, and vessels intact. Direct immunofluorescence showed linear deposit of IgG and smaller amounts of C3 in all capillary loops of the six glomeruli. Staining for IgA and IgM were negative. Electron microscopy was not performed.

A diagnosis of Goodpasture's syndrome was made and treatment with oxygen, blood transfusion, once every other day haemodialysis and plasmapheresis, with four litre exchanges and two units of fresh frozen plasma, three 1 g methylprednisolone boli, followed by prednisone 1 mg/kg/day and cyclophosphamide 750 mg/m^2^ of body surface area bolus with a 75% adjustment for renal failure (1000 mg/pulse) initiated. The patient denied haemoptysis or other respiratory symptoms after the second session of plasmapheresis; pulmonary sounds were normal and the anti-GBM antibodies titres became negative before the eleventh session. A total of twelve sessions were completed and the bolus of cyclophosphamide was repeated two weeks after the previous dose. Serum creatinine levels remained high, with mean predialytic value 7.1 mg/dL, as he evolved to anuria.

Three days after the twelfth plasmapheretic treatment the patient began shortness of breath, cough with haemoptysis, inspiratory rales and hypoxia. Fluid overload, restarting smoking or other respiratory aggression were ruled out. Coagulopathy was being prevented by the use of two units of fresh frozen plasma at the end of each pheresis treatment. Relapsing pulmonary haemorrhage with negative anti-GBM antibodies, part of Goodpasture's syndrome was assumed and treatment resumed: another three day boli of 1 g of methylprednisolone and daily plasmapheresis with four litre exchanges were started. After the two boli of cyclophosphamide (the last eleven days prior) it was decided to start mycophenolate mofetil 500 mg twice a day. On the second day the complaints ceased, but on the sixth day recrudescence of cough, haemoptysis, dyspnoea with hypoxaemic respiratory failure, and anaemia determined a brief intensive care unit stay for surveillance. The mycophenolate mofetil dose was doubled (1 g every 12 hours), daily plasmapheresis and oral prednisolone 1 mg/kg/day were maintained. He evolved with sustained recovery of pulmonary disturbances following the second day of the increased mycophenolate dosage. Despite favourable respiratory evolution, kidney function was lost, as creatinine levels remained high (mean predialytic value 6.7 mg/dL), maintaining anuria and dependence upon dialysis. This second course of plasmapheresis was stopped after sixteen sessions and the patient was discharged with resolution of lung haemorrhage and established chronic renal failure.

After one year of followup and since the start of the full dose of mycophenolate mofetil, no other episode of pulmonary haemorrhage has occurred, no pulmonary permanent lesions were found six months after and anti-GBM antibodies have been negative since the first course of plasmapheresis. The mycophenolate dose was decreased six months later to 500 mg every 12 hours, and steroid doses slowly reduced to 5 mg per day. As anti-GBM antibodies remain undetectable this patient is now on the active waiting list for renal transplant.

## 3. Discussion

Mycophenolate mofetil has been used since the 1990s as an immunosuppressive drug for the prevention of rejection in kidney transplantation. Due to favourable experience, with fewer adverse effects, it is currently used in liver, lung, and bone marrow transplantation and in the treatment of several autoimmune diseases, such as systemic lupus erythematosus, systemic vasculitis [7, 8] and some primary glomerular diseases [7, 9]. It suppresses the immune response by inhibiting activated lymphocytes, blocks purine synthesis, and inhibits specific proliferative responses of both T and B cells to antigens [8, 10]. Its effect on B cells can inhibit humoral immune response and antibody production [11], therefore stopping the autoimmune response to the triggering event and preventing lesion cascade. Other proposed mechanisms include the induction of apoptosis of activated T lymphocytes and the inhibition of adhesion molecule expression, which will lead to decreased recruitment of lymphocytes and monocytes into sites of inflammation [12].

To our knowledge this is the second report of the use of mycophenolate mofetil in the treatment of Goodpasture's syndrome [13]. Both cases had similar features of life-threatening disease, were refractory to conventional therapy and reported success with the use of this drug as second line treatment, with the dosage of 1 g every 12 hours [13]. In our experience the use of a lower dose mycophenolate mofetil regimen turned out to be insufficient, as in this patient only the dosage of 1 g every 12 hours induced remission of the pulmonary haemorrhage.

Although the scientific value of a single case could be questionable, randomised trials are almost impossible to conduct in such a rare disease. Based on the high mortality and current insufficient therapeutic weapons, in our opinion mycophenolate mofetil should be considered an alternative treatment, mainly in relapsing disease.

##  Conflict of Interests

The authors declared that there is no conflict of interest.
